# Interindividual Variability in Raffinose Fermentation by Human Gut Microbiota and Metabolomic Signature of a High-Efficiency In Vitro Degrader, *Limosilactobacillus reuteri* subsp. *reuteri*

**DOI:** 10.3390/foods15152649

**Published:** 2026-07-28

**Authors:** Wei Dai, Min Quan, Guangli Yu, Qingsen Shang

**Affiliations:** 1 Key Laboratory of Marine Drugs of Ministry of Education, Shandong Key Laboratory of Glycoscience and Glycotherapeutics, School of Medicine and Pharmacy, Ocean University of China, Qingdao 266003, China; daiwei3266@stu.ouc.edu.cn (W.D.); q15265991920@163.com (M.Q.); glyu@ouc.edu.cn (G.Y.); 2 Laboratory for Marine Drugs and Bioproducts, Qingdao Marine Science and Technology Center, Qingdao 266237, China; 3 Marine Biomedical Research Institute of Qingdao, Qingdao 266071, China

**Keywords:** raffinose, gut microbiota, prebiotic, probiotic, metabolism, *Limosilactobacillus reuteri*

## Abstract

**Background/Objectives**: Raffinose is a prebiotic trisaccharide fermented by human gut microbiota, yet the strain-level determinants of its degradation and associated metabolic outputs remain poorly characterized. **Methods**: In the present study, anaerobic fermentation, 16S rRNA gene amplicon high-throughput sequencing, culturomics, and metabolomics were conducted to address this question. **Results**: In vitro fermentation of raffinose was performed using fecal samples from 16 healthy donors, and substantial interindividual variation was observed in substrate consumption, microbiota composition, and short-chain fatty acid production. Through culturomics, 204 bacterial strains spanning 18 species were isolated, among which *Limosilactobacillus reuteri* subsp. *reuteri* exhibited the highest raffinose-degrading efficiency. Untargeted metabolomics comparing *L. reuteri* subsp. *reuteri* cultured on raffinose versus glucose revealed a distinct metabolic shift, with 290 metabolites significantly upregulated, including the putatively identified fructooligosaccharide 1-kestose—a compound with documented prebiotic and anti-inflammatory properties. KEGG enrichment further highlighted coordinated remodeling of nucleotide and amino acid metabolism. **Conclusions**: Collectively, this study provides a strain-level framework for understanding how raffinose shapes the gut microbiota and identifies *L. reuteri* subsp. *reuteri* as a key metabolic hub linking prebiotic consumption to the production of bioactive metabolites.

## 1. Introduction

**Figure 1:**
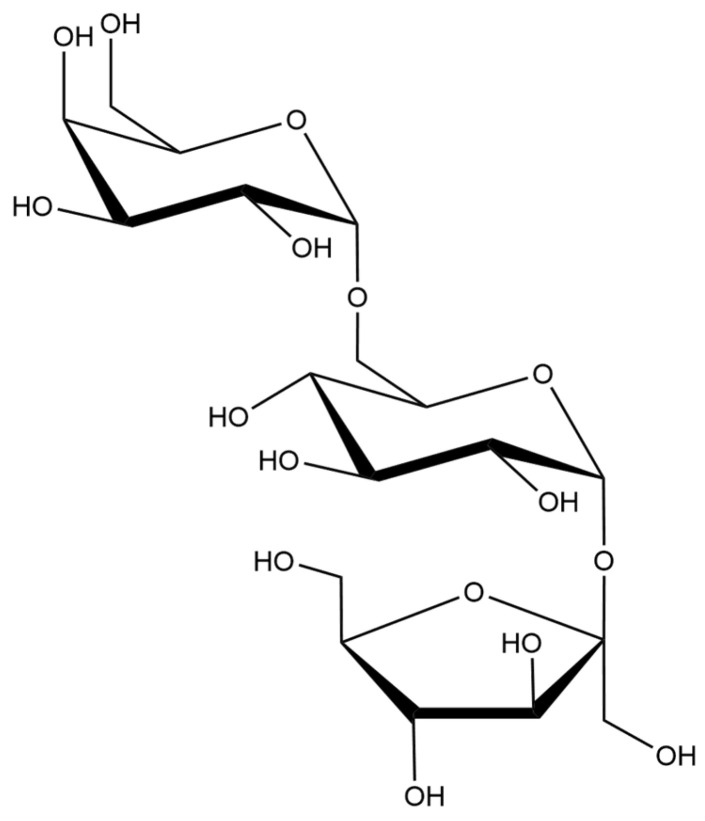
Chemical structure of raffinose.

Raffinose, a naturally occurring trisaccharide composed of galactose, glucose, and fructose (Figure 1), is abundantly present in legumes, grains, and various vegetables [1]. Unlike digestible carbohydrates, raffinose resists hydrolysis by host digestive enzymes due to the absence of endogenous α-galactosidase in humans, allowing it to reach the colon intact where it serves as a fermentable substrate for the gut microbiota [2,3]. Historically considered an anti-nutritional factor responsible for flatulence [1], raffinose has recently gained recognition as a promising prebiotic agent capable of modulating gut microbial composition and metabolic activity [2,4].

The human gut microbiota, comprising trillions of microorganisms, plays a critical role in extracting energy from otherwise indigestible dietary components and in producing a wide array of metabolites that influence host physiology [5,6,7]. Among these metabolites, short-chain fatty acids (SCFAs) such as acetate, propionate, and butyrate are key mediators of gut barrier function, immune regulation, and metabolic homeostasis [8,9]. Previous studies have demonstrated that raffinose fermentation can selectively promote the growth of beneficial taxa, particularly *Bifidobacterium* spp. and *Lactobacillus* spp., while stimulating SCFA production [2,3,4]. However, most of these studies have focused on a limited number of fecal donors, leaving the interindividual variability in raffinose degradation largely unexplored. In addition, these foundational studies date back to 2018–2020 [2,3], and the field has seen little subsequent progress on pure raffinose fermentation, particularly regarding strain-level degraders and their metabolic outputs. This knowledge gap prompted our comprehensive re-investigation using an integrated culturomics and metabolomics approach.

Emerging evidence highlights that the capacity to ferment specific prebiotics varies considerably across individuals, driven by differences in baseline gut microbiota composition [10,11,12]. Such interindividual variation has profound implications for personalized nutrition strategies, yet the strain-level determinants underlying differential raffinose utilization remain poorly understood [2,3,4]. Moreover, beyond SCFAs, the broader metabolic landscape generated by specific bacterial strains during raffinose fermentation, including potentially bioactive compounds, has not been systematically characterized.

Culturomics has recently proven effective in pinpointing functionally dominant degraders of other dietary components in the human gut, such as chondroitin sulfate [13], carrageenan [14], alginate [15], and the macromolecular protein Ejiao [16]. However, its application to raffinose—a prebiotic trisaccharide with well-documented interindividual responses—has not been reported. Unlike metagenomic predictions that infer function from gene content, culturomics enables direct phenotypic validation of substrate degradation and downstream metabolic output at the strain level, which is essential for establishing causality rather than correlation in host–microbiome interactions [13,14,15,16].

In the present study, we hypothesized that raffinose fermentation is highly personalized at the functional level, and that the most efficient raffinose-degrading strain may not be the most abundant community member, but rather a low-abundance specialist with a distinct metabolic output. To test this hypothesis, we established three specific objectives: (i) to quantify interindividual variability in raffinose consumption and SCFA production across 16 healthy donors; (ii) to isolate and functionally screen raffinose-degrading bacteria using culturomics; and (iii) to compare the global metabolomic profiles of the most efficient degrader cultured on raffinose versus glucose. To achieve these objectives, we employed an integrative approach combining in vitro anaerobic fermentation, 16S rRNA gene sequencing, culturomics, and untargeted metabolomics.

Our findings provide a strain-level framework for understanding the personalized nature of raffinose fermentation and identify *L. reuteri* subsp. *reuteri* (reclassified from *Lactobacillus reuteri* in 2020 based on phylogenetic and functional distinctiveness [17]) as a key bacterial player linking prebiotic consumption to the production of bioactive metabolites with potential health-promoting properties. Beyond mechanistic insights, this strain-level characterization has clear translational value. It provides a rational basis for developing a targeted synbiotic strategy that combines raffinose with *L. reuteri* subsp. *reuteri* R14-1. Such an approach could serve as a precision tool to modulate gut microbial metabolism and host inflammatory responses in future clinical or personalized nutrition settings.

## 2. Materials and Methods

### 2.1. Chemicals and Reagents

MRS medium (Cat. No. HB0384-1) was acquired from Hopebio (Qingdao, Shandong, China). L-cysteine hydrochloride (Cat. No. A600132-0100), agar (Cat. No. A600305-0500), and hemin chloride (Cat. No. A602521-0025) were obtained from Sangon Biotech (Shanghai, China). Raffinose was sourced from Aladdin (Shanghai, China). Phosphate-buffered saline (PBS) was purchased from the Servicebio Technology (Wuhan, Hubei, China). Pre-coated thin layer chromatography (TLC) sheets (Cat. No. 818233) used in the present study were obtained from MACHEREY-NAGEL Gmbh & Co. KG (Düren, Nordrhein-Westfalen, Germany).

Tween 80, peptone, yeast extract, and tryptone were purchased from Sigma-Aldrich (St. Louis, MO, USA). All other chemicals, including KOH, NaCl, KCl, ZnSO_4_·7H_2_O, MgSO_4_·7H_2_O, FeSO_4_·7H_2_O, CaCl_2_·2H_2_O, MnCl_2_·4H_2_O, CoCl_2_·H_2_O, KH_2_PO_4_, CuSO_4_·5H_2_O, NiCl_2_·6H_2_O, H_2_SO_4_, and CH_3_CH_2_OH were procured from Sinopharm Chemical Reagent (Shanghai, China), unless otherwise stated.

### 2.2. Collection of Human Fecal Samples

A total of 16 healthy volunteers (7 males and 9 females) were enrolled in this study (Figure S1). The participant cohort consisted of one 4-year-old girl, with the remaining participants being young adults aged 23 to 26 years (Figure S1). None of the participants had taken antibiotics, probiotics, or prebiotics for at least six months prior to sample collection. All donors were residents of Qingdao, China, and were therefore considered to share similar geographic and dietary backgrounds. Detailed information on body mass index and habitual dietary fiber intake was not systematically collected, as this study was designed as an exploratory functional investigation with a primary focus on microbial strain-level characterization rather than host–covariate association analysis. All participants have signed the informed consent. The study protocol was approved by the Ethical Committee of Marine Biomedical Research Institute of Qingdao (E-MBMT-2023-4-1, approved on 1 April 2023).

A sample size of 16 donors was chosen based on the feasibility of the subsequent labor-intensive culturomics and metabolomic analyses, and was deemed sufficient to detect pronounced interindividual variation based on previous similar in vitro fermentation studies [13,14,15,16]. No formal power calculation was performed, as this was an exploratory functional study rather than a hypothesis-testing clinical trial. The fresh fecal samples were carefully collected into a sterile excrement collector. After that, the samples were immediately transferred into an Electrotek AW 500SG anaerobic (80% N_2_, 10% H_2_, and 10% CO_2_) chamber (Shipley, West Yorkshire, UK).

### 2.3. Batch Fermentation of Raffinose

The fecal samples were homogenized with anaerobic PBS to prepare a 20% (wt/vol) bacterial suspension. Food residues and insoluble substances in the fecal samples were removed by slowly passing the slurries through a 0.4 mm sieve. Then, 1 mL of the suspension was inoculated into anaerobic VI medium and incubated at 37 °C. The remaining fecal samples were stored at −80 °C for subsequent analysis. The VI medium was used for the in vitro anaerobic fermentation as previously described [13,14,15]. The fermentation experiments were conducted in the Electrotek AW 500SG anaerobic (80% N_2_, 10% H_2_, and 10% CO_2_) chamber (Shipley, West Yorkshire, UK). All batch fermentations were performed in sterile serum bottles sealed with butyl rubber stoppers and caps to maintain strict anaerobic conditions.

The VI medium contained the following components: raffinose, 8.0 g/L; peptone, 3.0 g/L; tryptone, 3.0 g/L; yeast extract, 4.5 g/L; hemin chloride, 0.05 g/L; NaCl, 4.5 g/L; KCl, 2.5 g/L; MgSO_4_·7H_2_O, 4.5 g/L; CaCl_2_·6H_2_O, 0.2 g/L; KH_2_PO_4_, 0.4 g/L; L-cysteine hydrochloride, 0.8 g/L; Tween 80, 1 mL/L; and trace elements solution, 0.2 mL/L. The trace elements solution contained the following chemicals: MgSO_4_·7H_2_O, 3.0 g/L; CuSO_4_·5H_2_O, 0.01 g/L; FeSO_4_·7H_2_O, 0.1 g/L; ZnSO_4_·7H_2_O, 0.18 g/L; CoSO4·7H_2_O, 0.18 g/L; CaCl_2_·2H_2_O, 0.1 g/L; MnCl_2_·4H_2_O, 0.32 g/L; NiCl_2_·6H_2_O, 0.092 g/L. Raffinose was added to the medium as the sole added fermentable carbohydrate.

### 2.4. Carbohydrate Utilization Analysis

To assess raffinose degradation by human fecal microbiota, TLC was performed as described previously [13,14,15]. The fermentation medium was collected at 0 h, 12 h, 24 h, 36 h and 48 h. After centrifugation, a 0.4 µL aliquot of each supernatant was loaded onto the pre-coated TLC sheet. The plate was developed with a freshly prepared mixture of formic acid, *n*-butanol, and water (6:4:1, *v*/*v*/*v*) until the solvent front migrated to 5 mm from the top edge, after which it was removed and air-dried. To achieve better separation of different carbohydrate components, a second development was performed using the same solvent system. The plate was then immersed in orcinol-sulfuric acid reagent, and the spots were visualized by heating at 105 °C for 2–3 min in the oven from Yiheng Scientific Instruments (Shanghai, China).

Additionally, raffinose consumption throughout the fermentation process was monitored using the phenol–sulfuric acid method, as described previously [13,14,15]. The fermentation medium was collected at 0 h, 12 h, 24 h, 36 h, and 48 h. After centrifugation, 25 µL of each supernatant was transferred to glass test tubes and diluted with distilled water to a final volume of 200 µL. An equal volume of 6% (*w*/*v*) phenol was then added, followed by 1.5 mL of concentrated H_2_SO_4_. After the solution turned orange-yellow, it was mixed thoroughly and placed in a water bath at 100 °C for 10 min. The solution was then cooled in cold water, and 200 µL was taken for absorbance measurement at 495 nm using the microplate spectrophotometer from Flash Spectrum Biological Technology (Shanghai, China).

### 2.5. SCFAs Analysis

Approximately 1 mL of the fermentation culture was collected at 0 h, 12 h, 24 h, 36 h and 48 h and each sample was mixed with an equal volume of 1% (vol/vol) H_2_SO_4_. Subsequently, 200 µL of the mixed liquid was filtered through a 0.22-μm membrane filter, and the filtrate was transferred into a liquid chromatography vial for analysis. SCFAs generated during raffinose fermentation were quantified using an Agilent 1260 high-performance liquid chromatography (HPLC) system (Santa Clara, CA, USA) as previously described [13,14,15]. The mobile phase consisted of 5 mM sulfuric acid applied isocratically at a flow rate of 0.6 mL/min, with the column temperature maintained at 50 °C and the detection wavelength set at 210 nm.

### 2.6. 16S rRNA Gene Amplicon High-Throughput Sequencing and Bioinformatics Analysis

The fresh fecal samples were collected from 16 healthy volunteers, with approximately 80 mg of each sample taken for DNA extraction. Bacterial genomic DNA was extracted using the SPINeasy DNA Kit for Feces from MP Biomedicals (Solon, OH, USA) following the manufacturer’s instructions. Bacterial genomic DNA was extracted from both the human fecal samples and the post-fermentation bacterial suspensions. DNA concentration and purity were assessed using a NanoDrop 2000 spectrophotometer from Thermo Fisher Scientific (Waltham, MA, USA). The V3–V4 hypervariable region of the bacterial 16S rRNA gene was amplified using universal primers 338F and 806R. The 16S rRNA gene amplicons were sequenced using the Illumina PE300 platform (San Diego, CA, USA) from Majorbio Bio-Pharm Biotechnology (Shanghai, China). Bioinformatic analyses of the sequencing data, including α-diversity analysis, β-diversity analysis, and Wilcoxon rank-sum test analysis were all conducted using online tools from Majorbio Cloud Platform [18].

### 2.7. Isolation of Raffinose-Degrading Bacteria

Approximately 2 g of fecal sample was placed in an autoclaved 10 mL centrifuge tube, and sterile double-distilled water was added. The mixture was vortexed to suspend the microorganisms, yielding a fecal bacterial suspension. After a brief settling period, the suspension was filtered through a 0.4 mm metal sieve to remove large insoluble particles and debris. The bacterial suspension was then serially diluted with sterile double-distilled water to concentrations ranging from 10^−1^ to 10^−6^. Aliquots (100 µL) of three dilutions (10^−4^, 10^−5^, and 10^−6^) were spread onto solid VI medium containing raffinose as the sole added fermentable carbohydrate. Once colonies reached a suitable size, single colonies were picked and purified by streaking using the two-zone streak plate method.

After two or three rounds of streak purification, individual colonies were transferred into liquid VI medium containing raffinose as the sole added fermentable carbohydrate using an inoculating loop and further cultured. All incubations were performed at 37 °C in the Electrotek AW 500SG anaerobic (80% N_2_, 10% H_2_, and 10% CO_2_) chamber (Shipley, West Yorkshire, UK). For long-term storage, a preservation solution consisting of 30% glycerol in double-distilled water was prepared and sterilized. An equal volume of bacterial suspension was added to the preservation solution to achieve a final glycerol concentration of 15%, and the mixture was stored at −80 °C.

### 2.8. Taxonomic Identification and Phylogenetic Tree Analysis

The 16S rRNA gene of the isolates were amplified with universal primers 27F and 1492R. Sequencing was performed using the Thermo Fisher Scientific (Waltham, MA, USA) 3730xl DNA Analyzer from Sangon Biotech (Shanghai, China) as previously described [13,14,15]. Only sequences between 30 bp and 750 bp were considered reliable for further analysis. High-quality sequences were analyzed using Chromas (Version 2.6.6). The resulting sequences were compared with the EzBioCloud database for taxonomic identification as detailed elsewhere [19]. Phylogenetic tree analysis of the raffinose-degrading bacteria was conducted using maximum likelihood method from the Molecular Evolutionary Genetics Analysis (MEGA) software (version 7.0.26).

### 2.9. Metabolomic Analysis

*L. reuteri* subsp. *reuteri* R14-1 was revived using MRS medium. For metabolomic analysis, the strain was cultured in VI medium supplemented with either glucose (Glc) or raffinose (Raf) at a concentration of 8.0 g/L as the sole added fermentable carbohydrate. Glucose medium served as the control, and raffinose medium served as the experimental group. When the strain reached the optimal growth state after approximately 24 h of cultivation, it was transferred to 50 mL of the respective medium and incubated anaerobically at 37 °C in the Electrotek AW 500SG anaerobic (80% N_2_, 10% H_2_, and 10% CO_2_) chamber (Shipley, West Yorkshire, UK). Six independent biological replicates were prepared for each condition. Bacterial growth was monitored by measuring optical density at 600 nm (OD_600_), and carbohydrate degradation was assessed by TLC using samples collected every 2 h. Fermentation was terminated upon complete consumption of the respective carbon sources.

The fermentation broth was centrifuged at 12,000 rpm for 10 min to collect the bacterial pellet. The pellet was washed three times with PBS to remove residual medium components, resuspended in PBS, and aliquoted into six 1.5 mL microcentrifuge tubes. Following a final centrifugation step, the supernatant was discarded, and the pellets were flash-frozen in liquid nitrogen for at least 5 min, then stored at −80 °C until further analysis.

Mass spectrometry (MS) data were acquired in both negative and positive ion modes. Raw MS data were preprocessed using Progenesis QI software (v. 2.0) from Waters Corporation (Milford, CT, USA). Metabolite identification was performed by searching against the Human Metabolome Database, METLIN, and the Majorbio Database. Subsequent data processing and analysis, including principal component analysis (PCA), partial least squares-discriminant analysis (PLS-DA), Venn diagram analysis, Volcano plot analysis, KEGG pathway analysis were all conducted with the help of online tools from Majorbio Cloud Platform [19]. Metabolites with variable importance in projection (VIP) > 1 and a *p* value < 0.05 (Student’s *t*-test) were considered significantly differentially abundant.

### 2.10. Statistical Analysis

All data are presented as mean ± standard error of the mean (SEM). Statistical comparisons between two groups were performed using Student’s *t* test, while comparisons among multiple groups were conducted using one way analysis of variance (ANOVA) followed by Tukey’s post hoc test. All analyses were carried out using GraphPad Prism (version 8.0.2) (San Diego, CA, USA).

For metabolomic data analysis, to correct for multiple comparisons across all detected metabolites, *p* values were adjusted using the false discovery rate (FDR) method of Benjamini–Hochberg. Metabolites with FDR-adjusted *p* < 0.05 were considered statistically significant. Partial least squares discriminant analysis (PLS-DA) was performed to visualize global metabolic differences between groups and to identify metabolites contributing to group separation. Metabolites with Variable Importance in Projection (VIP) scores > 1.0 were considered important contributors to group discrimination. Metabolites meeting both criteria (VIP > 1.0 and FDR-adjusted *p* < 0.05) were defined as significantly differential metabolites and subjected to KEGG pathway enrichment analysis.

## 3. Results

### 3.1. Individual Variation in Raffinose Fermentation and SCFAs Production by Human Gut Microbiota

**Figure 2:**
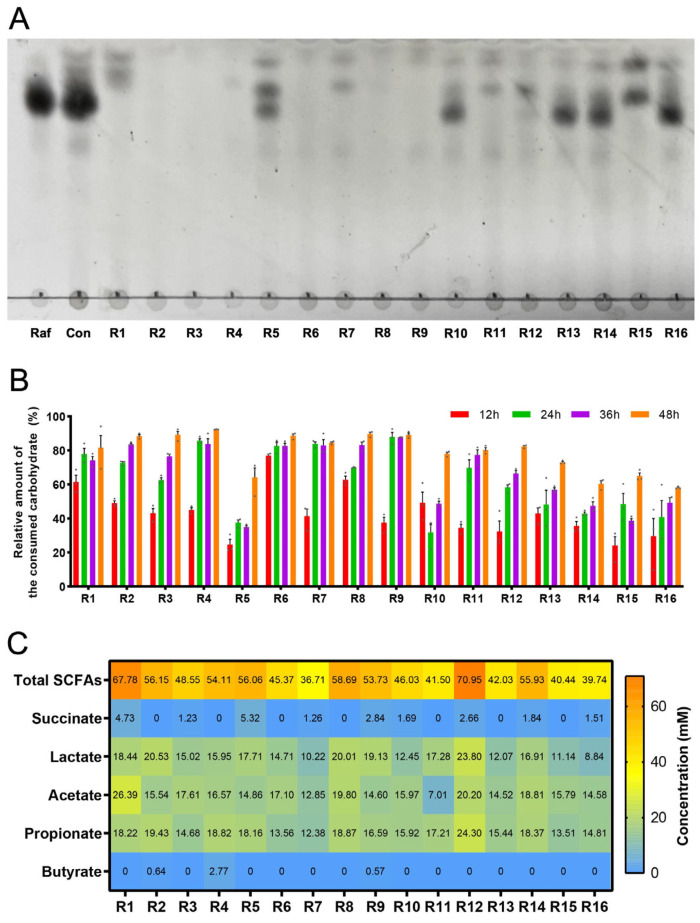
In vitro fermentation of raffinose by human gut microbiota from 16 fecal samples. TLC analysis of raffinose degradation (**A**). Relative amount of raffinose consumed at different time points (**B**). SCFA production (**C**).

To investigate the fermentation characteristics of raffinose, fresh fecal samples were collected from 16 healthy volunteers and inoculated into separate VI medium containing raffinose as the sole added fermentable carbohydrate. Substantial interindividual variation was observed in both substrate degradation and metabolic output (Figure 2). TLC analysis revealed that raffinose was completely degraded by the microbiota from six donors (R2, R3, R4, R6, R8, and R9), whereas the remaining samples exhibited only partial or limited degradation capacity (Figure 2A). Quantitative assessment of raffinose consumption throughout the fermentation period corroborated these findings, with the same six donors showing extensive substrate utilization (Figure 2B).

SCFAs production in each culture was subsequently quantified. The highest total SCFAs concentration was observed in sample R12, followed by R1 (Figure 2C). Notably, the donors that achieved complete raffinose degradation (R2, R3, R4, R6, R8, and R9) were not those that produced the highest SCFAs levels, indicating that substrate consumption does not directly predict metabolic output. Collectively, although raffinose was fermentable by the gut microbiota across all 16 samples, both degradation efficiency and SCFAs production exhibited marked interindividual variation, underscoring the personalized nature of prebiotic utilization.

### 3.2. Structural Shifts in Gut Microbiota Following Raffinose Fermentation

**Figure 3:**
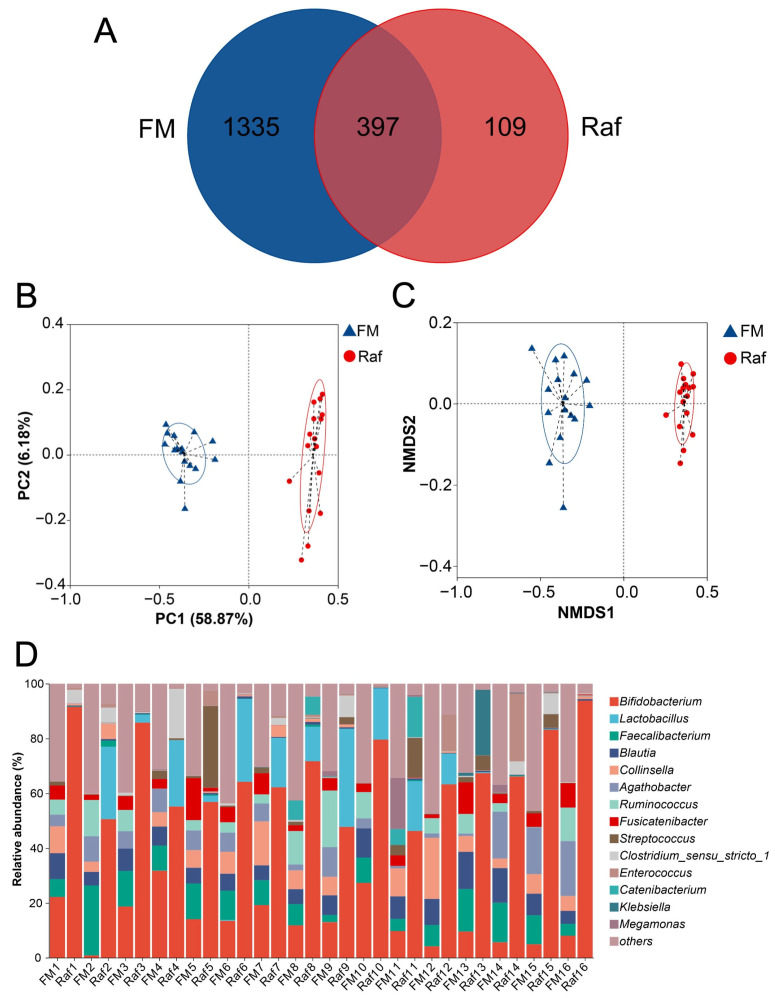
Composition and structure of gut microbiota before and after raffinose fermentation. Venn diagram showing shared and unique operational taxonomic units (OTUs) (**A**). PCoA analysis (**B**). NMDS analysis (**C**). Microbial composition at the genus level (**D**).

Changes in gut microbiota composition following raffinose fermentation were investigated using 16S rRNA gene sequencing. Analysis of α-diversity indices (Simpson, Shannon, Chao1, and Ace) revealed a significant reduction in microbial diversity after fermentation compared to baseline (Figure S1), suggesting selective enrichment of bacterial taxa capable of utilizing raffinose. β-Diversity analysis, assessed by Venn diagrams, principal coordinate analysis (PCoA), and non-metric multidimensional scaling (NMDS), further demonstrated that the overall structure of the fecal microbiota was significantly altered post-fermentation (Figure 3A–C). Community composition analysis at the genus level corroborated these shifts (Figure 3D).

**Figure 4:**
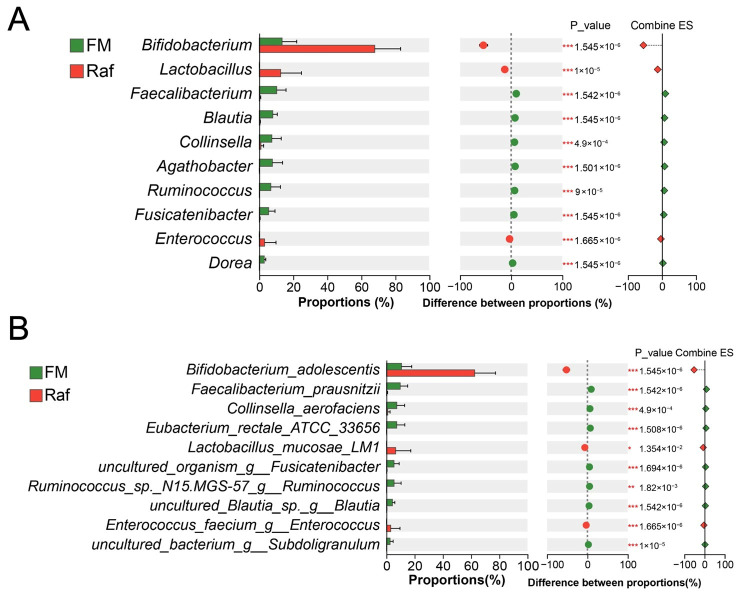
Comparative analysis of gut microbiota before and after in vitro raffinose fermentation. Wilcoxon rank-sum test at the genus (**A**) and species (**B**) levels. * *p* < 0.05, ** *p* < 0.01, *** *p* < 0.001.

To identify bacterial taxa enriched by raffinose fermentation, Wilcoxon rank-sum tests analysis were performed (Figure 4). The relative abundances of *Bifidobacterium* spp. and *Lactobacillus* spp. were significantly increased following raffinose fermentation (Figure 4A). At the species level, *Bifidobacterium adolescentis* and *Lactobacillus mucosae* were consistently enriched, whereas the abundances of *Faecalibacterium prausnitzii*, *Collinsella aerofaciens*, and *Eubacterium rectale* were significantly decreased (Figure 4B). These compositional changes indicate that raffinose selectively promotes the growth of specific beneficial genera while suppressing other commensal taxa.

### 3.3. Isolation of Raffinose-Degrading Bacteria from Human Gut Microbiota

**Figure 5:**
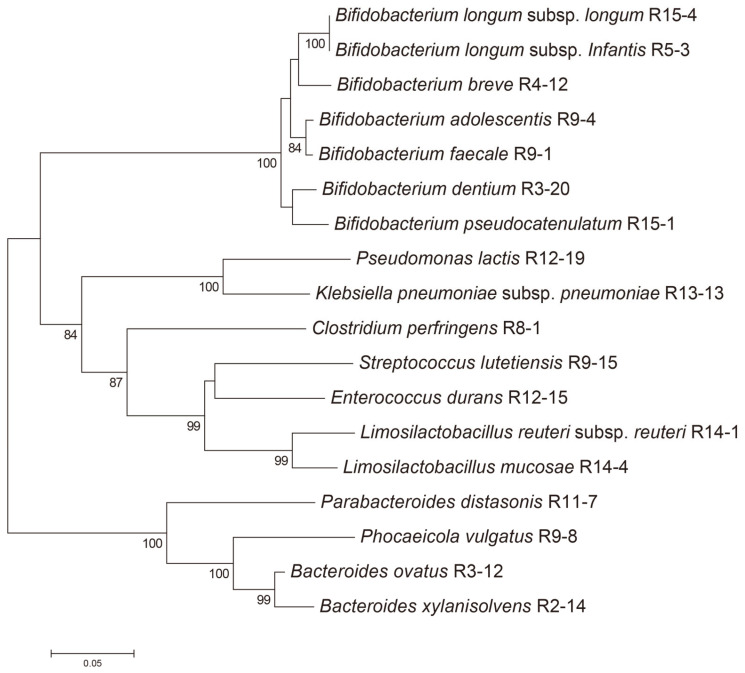
Phylogenetic tree of raffinose-degrading bacterial strains based on 16S rRNA gene sequences.

To identify bacterial taxa responsible for raffinose utilization, culturomics analysis was performed on the 16 fecal samples. A total of 204 bacterial strains representing 18 distinct species were isolated (Figure S2). One representative strain from each species was selected, and a phylogenetic tree was constructed based on 16S rRNA gene sequences to illustrate their evolutionary relationships (Figure 5).

**Figure 6:**
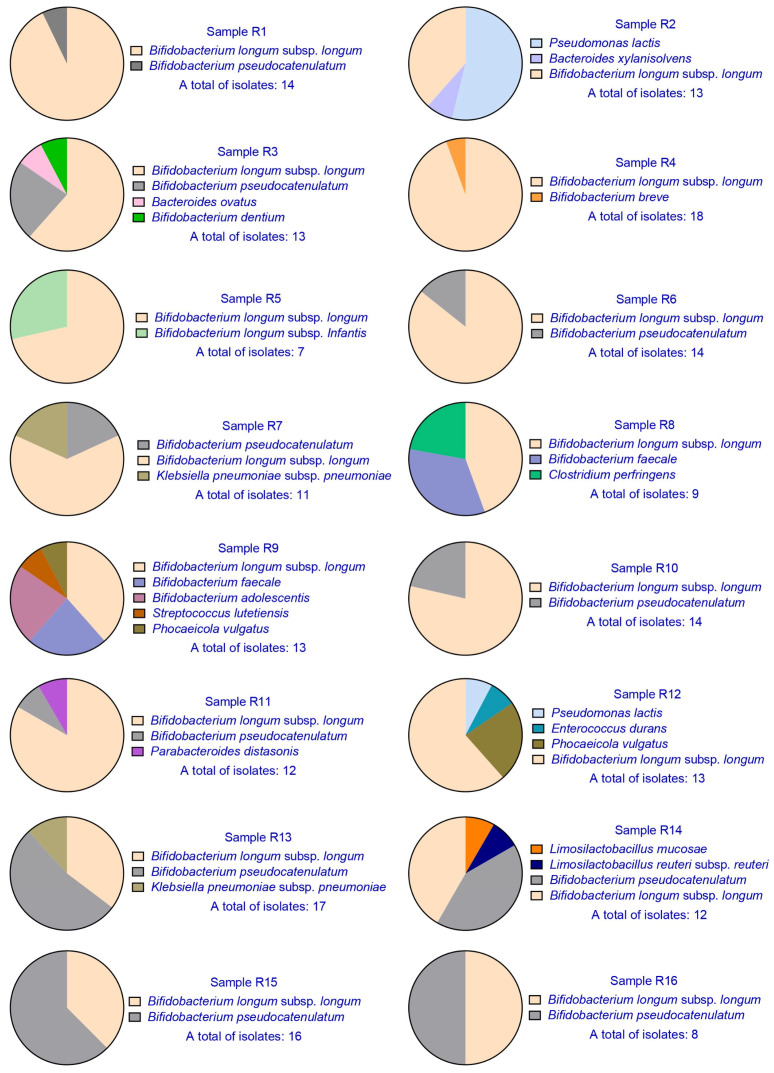
Abundance of raffinose-degrading bacterial strains isolated from each human fecal sample.

The relative abundance of isolated strains was further quantified across the 16 fecal samples. *Bifidobacterium* spp. exhibited the highest abundance among all isolates, consistent with their enrichment observed following raffinose fermentation (Figure 6 and Figure S2). This finding corroborated the enrichment patterns detected at the community level by 16S rRNA sequencing, suggesting that *Bifidobacterium* spp. are numerically dominant in the culturable raffinose-associated microbiota.

**Figure 7:**
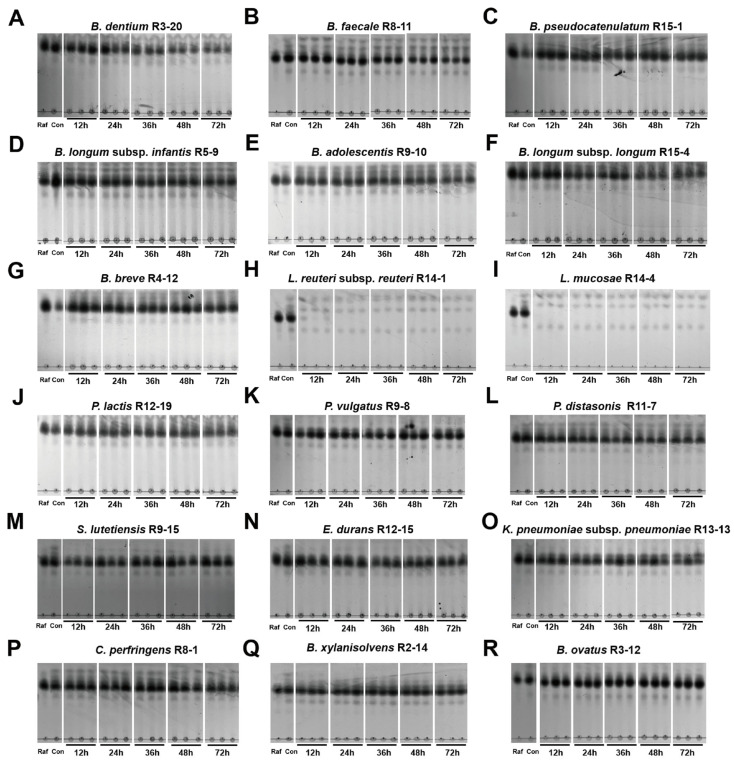
TLC analysis of raffinose degradation by 18 representative strains (**A**–**R**) over time.

The raffinose-degrading capacity of the 18 representative strains was evaluated by culturing each individually in VI medium containing raffinose as the sole added fermentable carbohydrate. These strains were selected to represent the diversity of raffinose-associated bacteria isolated from the 16 fecal samples. Culture supernatants were analyzed by TLC, which identified *L. reuteri* subsp. *reuteri* R14-1 and *Lactobacillus mucosae* R14-4 as the most efficient degraders (Figure 7). Notably, the TLC profiles revealed that these two strains almost completely degraded raffinose within 12 h, whereas most other strains showed minimal or no detectable degradation (Figure 7).

**Figure 8:**
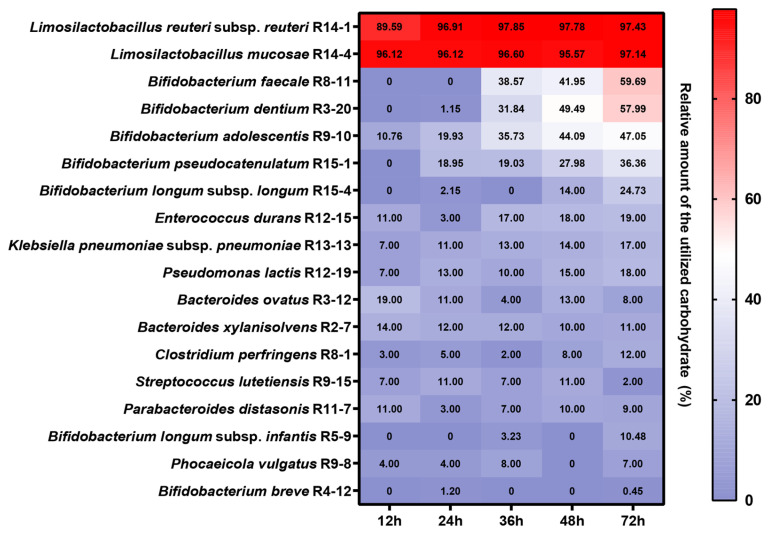
Relative amount of raffinose consumed by 18 representative strains at different time points.

Quantitative assessment of raffinose consumption across the 18 strains corroborated these findings, confirming that *L. reuteri* subsp. *reuteri* R14-1 and *L. mucosae* R14-4 consumed the highest amounts of substrate (Figure 8). Notably, despite the significant enrichment of *Bifidobacterium* spp. following raffinose fermentation, individual *Bifidobacterium* strains exhibited only limited capacity to utilize this trisaccharide, suggesting that other taxa may play a more direct role in its degradation. This functional discrepancy between abundance and activity highlights the importance of strain-level functional characterization beyond compositional analysis, and it further supports the selection of *L. reuteri* subsp. *reuteri* R14-1 and *L. mucosae* R14-4 for subsequent investigation.

### 3.4. Comparative Fermentation Profiles of L. reuteri subsp. reuteri R14-1 and L. mucosae R14-4 on Raffinose

Given that both *L. reuteri* subsp. *reuteri* R14-1 and *L. mucosae* R14-4 exhibited strong raffinose-degrading activity, their fermentation characteristics were further characterized under anaerobic conditions. TLC analysis confirmed that both strains almost completely degraded raffinose within 12 h (Figure 7H,I). To compare their growth and metabolic outputs, bacterial growth was monitored by optical density at 600 nm (OD_600_), SCFA production, and colony-forming unit (CFU) counts.

**Figure 9:**
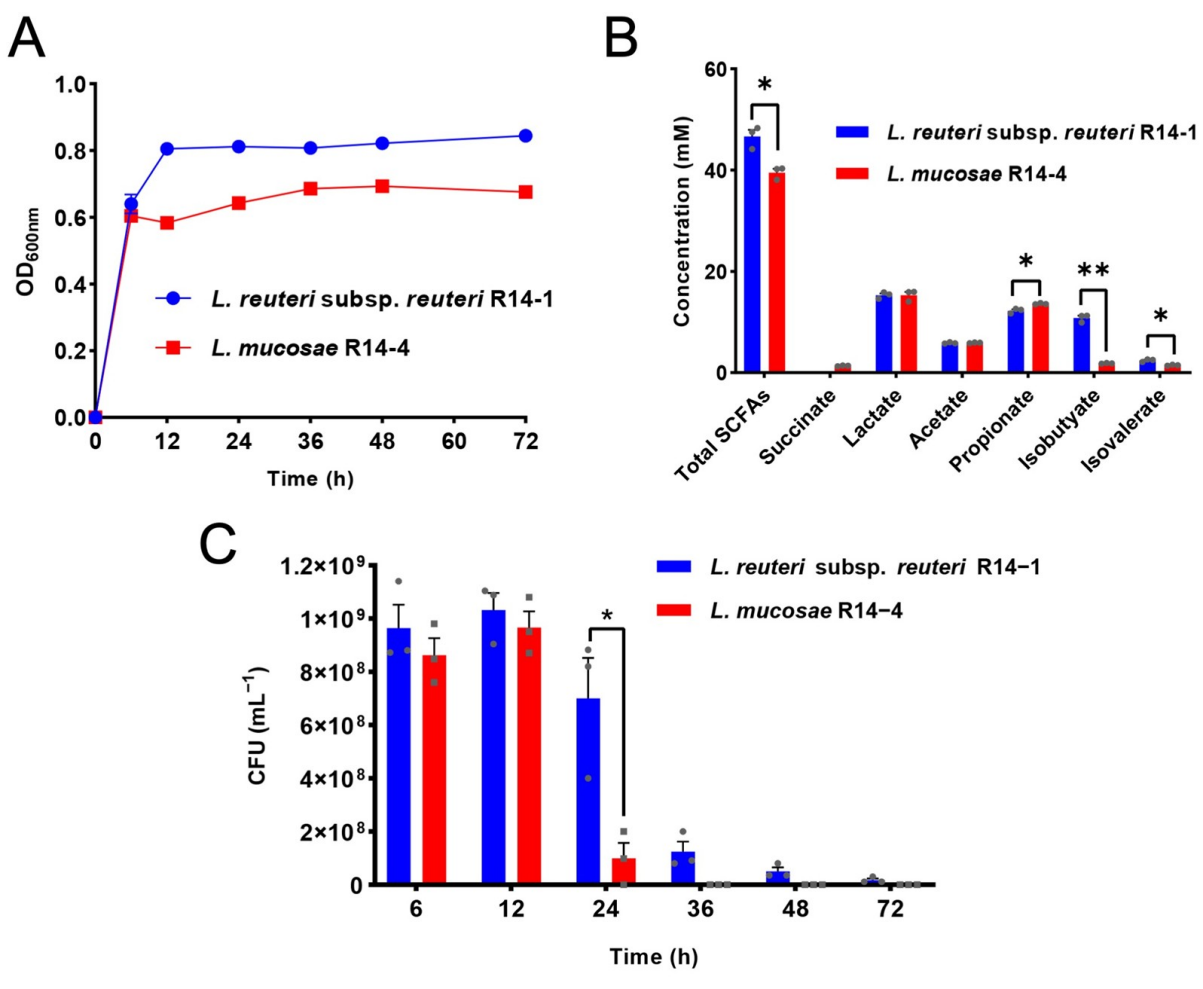
Comparison of growth and fermentation of raffinose by *L. reuteri* subsp. *reuteri* R14-1 and *L. mucosae* R14-4. Growth curves of the two strains in raffinose medium (**A**). Concentrations of SCFAs produced by the two strains (**B**). CFU counts of the two strains (**C**). * *p* < 0.05, ** *p* < 0.01.

*L. reuteri* subsp. *reuteri* R14-1 demonstrated superior growth performance compared to *L. mucosae* R14-4, as evidenced by higher OD_600_ values, greater total SCFA concentrations, and increased CFU counts throughout the fermentation period (Figure 9A–C). These results indicated that *L. reuteri* subsp. *reuteri* R14-1 was more efficient in utilizing raffinose for biomass accumulation and metabolite production, warranting further investigation into its metabolic output.

### 3.5. Global Metabolic Shift in L. reuteri subsp. reuteri R14-1 Cultured on Raffinose

**Figure 10:**
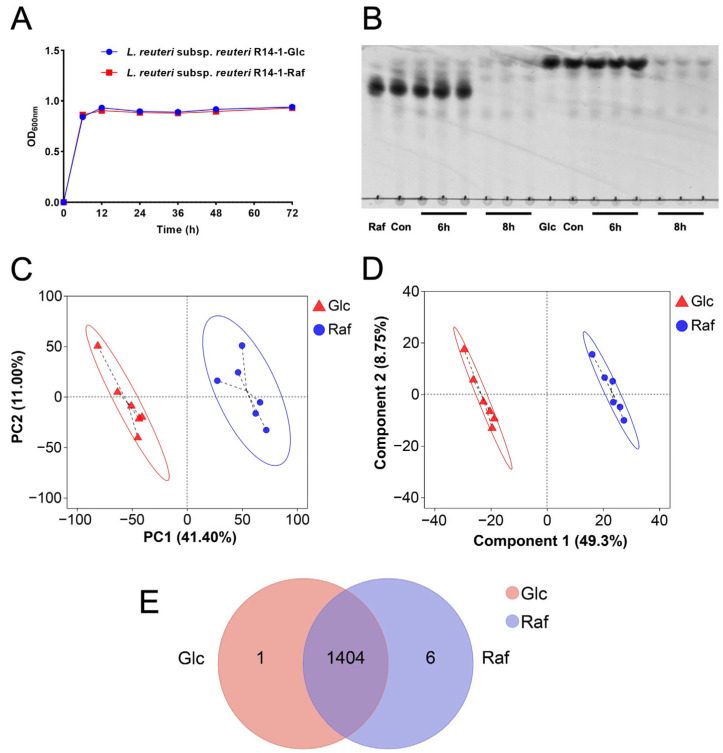
Comparison of metabolites produced by *L. reuteri* subsp. *reuteri* R14-1 cultured in raffinose versus glucose medium. Growth curves of *L. reuteri* subsp. *reuteri* R14-1 in raffinose and glucose media (**A**). TLC analysis of carbohydrate degradation by *L. reuteri* subsp. *reuteri* R14-1 in raffinose and glucose substrates (**B**). PCA (**C**) and PLS-DA analysis (**D**) of metabolites in the raffinose and glucose groups. Venn diagram analysis of metabolites in the raffinose and glucose groups (**E**).

To identify the specific metabolites produced by *L. reuteri* subsp. *reuteri* R14-1 during raffinose fermentation, comparative metabolomics was performed using VI medium supplemented with either raffinose or glucose as the sole added fermentable carbohydrate. Growth curve and TLC analyses confirmed that the strain grew to comparable densities on both substrates and completely consumed the respective carbohydrates within 8 h (Figure 10A,B), enabling a direct comparison of metabolic profiles under equivalent growth conditions.

Multivariate statistical analysis revealed distinct metabolic signatures between the two culture conditions. PCA and PLS-DA showed clear separation of the raffinose and glucose groups (Figure 10C,D), indicating a substantial shift in the metabolic landscape. Venn diagram analysis identified six unique metabolites associated with raffinose fermentation, whereas only one unique metabolite was detected in the glucose group (Figure 10E), suggesting that raffinose promotes a more diverse and specialized metabolic output.

**Figure 11:**
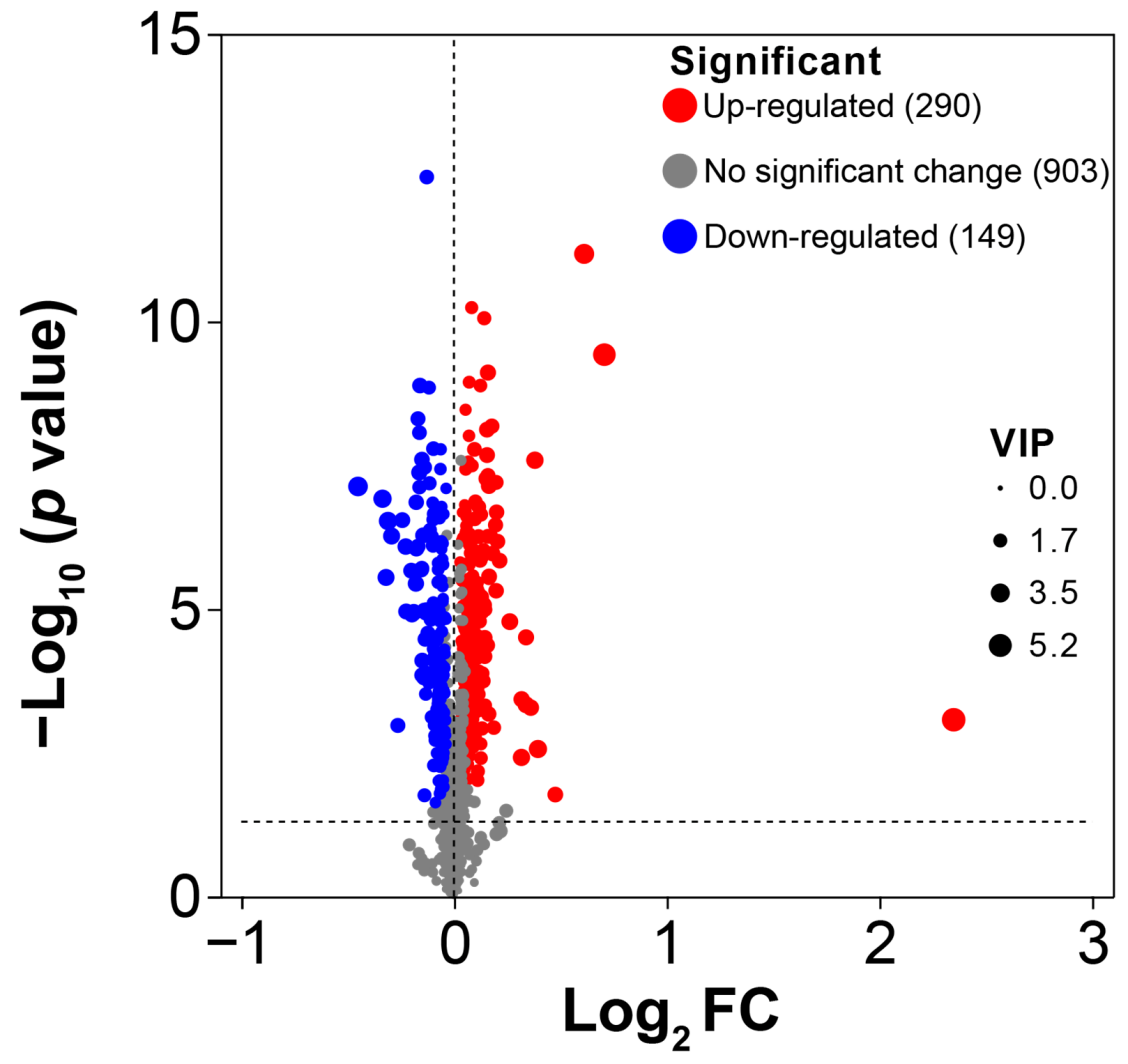
Volcano plot of differentially abundant metabolites produced by *L. reuteri* subsp. *reuteri* R14-1 cultured in raffinose versus glucose medium.

A total of 439 metabolites were significantly altered in the raffinose group compared to the glucose group (FDR < 0.05), of which 290 were up-regulated and 149 down-regulated (Figure 11). Notably, the concentrations of oxyphencyclimine and 1-kestose—both recognized for their anti-inflammatory and antioxidant properties—were significantly elevated in the raffinose group [20,21,22]. This observation suggests that raffinose fermentation by *L. reuteri* subsp. *reuteri* R14-1 may contribute to the production of bioactive metabolites with potential health-promoting functions.

### 3.6. Pathway Enrichment Reveals Coordinated Metabolic Remodeling of L. reuteri subsp. reuteri R14-1 in Response to Raffinose

**Figure 12:**
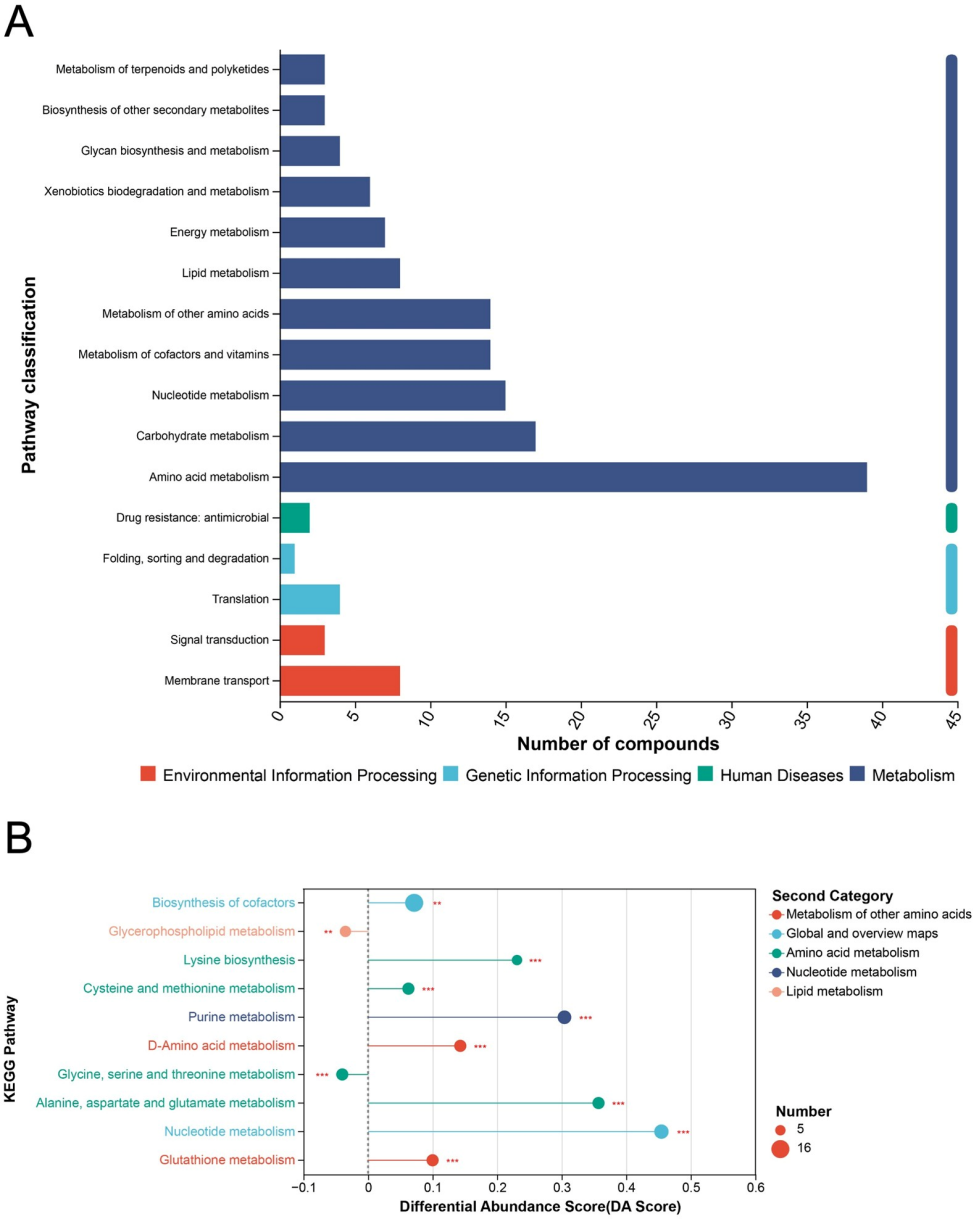
KEGG pathway analysis of metabolites produced by *L. reuteri* subsp. *reuteri* R14-1 cultured in raffinose versus glucose medium. KEGG pathway classification of differentially abundant metabolites (**A**). Bubble plot of enriched KEGG pathways (**B**). ** *p* < 0.01, *** *p* < 0.001.

KEGG pathway enrichment analysis of differentially abundant metabolites revealed that amino acid metabolism represented the most highly enriched functional category (Figure 12A). Specifically, pathways involved in nucleotide metabolism, alanine, aspartate and glutamate metabolism, purine metabolism, lysine biosynthesis, D-amino acid metabolism, and glutathione metabolism were significantly enriched in the raffinose group compared to the glucose group (Figure 12B). These pathways are intricately linked to bacterial growth, stress responses, and the biosynthesis of bioactive compounds.

Collectively, these findings indicate that raffinose not only serves as a fermentable carbon source but also orchestrates coordinated metabolic remodeling in *L. reuteri* subsp. *reuteri* R14-1. The observed enrichment of glutathione metabolism and amino acid-related pathways aligns with the elevated levels of anti-inflammatory metabolites (oxyphencyclimine and 1-kestose) [20,21,22], further supporting the functional relevance of this metabolic shift. Thus, raffinose appears to promote a distinct metabolic state in this strain that may contribute to its probiotic potential.

## 4. Discussion

**Figure 13:**
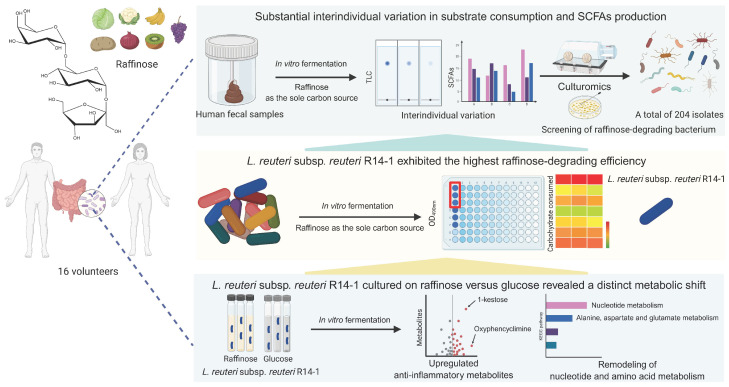
Summary of raffinose fermentation by human gut microbiota and metabolic reprogramming of *L. reuteri* subsp. *reuteri*. The figure was created with BioRender.com.

The human gut microbiota serves as a dynamic metabolic interface between diet and host physiology, with its composition and functional capacity exhibiting considerable interindividual variation [7,23,24,25]. In this study, we systematically dissected the fermentation characteristics of raffinose, a prebiotic trisaccharide, by integrating in vitro anaerobic fermentation, 16S rRNA gene sequencing, culturomics, and untargeted metabolomics (Figure 13). Our findings reveal that raffinose degradation is highly personalized, with substantial donor-dependent variation in both substrate consumption and SCFA production. Through strain-level functional characterization, we identified *L. reuteri* subsp. *reuteri* R14-1 as a key degrader capable of converting raffinose into a distinct set of bioactive metabolites, including the anti-inflammatory compounds oxyphencyclimine and 1-kestose. Collectively, this study establishes a strain-resolution framework for understanding the prebiotic mechanisms of raffinose and highlights the potential of *L. reuteri* as a partner in synbiotic strategies.

A notable observation in this study was the discordance between raffinose degradation efficiency and SCFA production across individual fecal samples. While complete substrate consumption was observed in six donors (R2, R3, R4, R6, R8, and R9), the highest total SCFA concentrations were detected in other samples (R12 and R1). This uncoupling suggests that substrate utilization alone does not dictate metabolic output; rather, the specific composition and metabolic capabilities of the resident microbiota determine the end products of fermentation. Consistent with this notion, we observed a significant decrease in the relative abundances of two butyrate-producing species, *F. prausnitzii* and *E. rectale* [26,27], following raffinose fermentation. Previous studies have shown that neither species utilizes lactate for butyrate production [28], which may explain their reduced abundance in raffinose-enriched cultures.

Additionally, the composition of the culture medium itself may also influence fermentation pathways and SCFA profiles. The VI medium used in this study contains peptone, tryptone, and yeast extract—sources of peptides and amino acids that can be co-metabolized alongside carbohydrates by gut bacteria. In complex media, microorganisms often shift toward mixed-acid fermentation or amino acid catabolism, which can alter the relative proportions of SCFAs (especially branched-chain fatty acids derived from amino acid fermentation) and dilute the quantitative relationship between carbohydrate consumption and SCFA production. Thus, while raffinose serves as the sole added carbohydrate, the presence of proteinaceous substrates in the basal medium may support alternative metabolic routes that decouple substrate degradation from canonical SCFA outputs. This medium effect, combined with interindividual differences in the metabolic preferences of the resident microbiota, further complicates direct predictions of SCFA production from degradation efficiency alone. Altogether, our findings underscore the functional complexity of the gut microbial ecosystem, where cross-feeding interactions and metabolic interdependencies shape overall SCFA profiles.

The selective enrichment of *Bifidobacterium* spp. and *Lactobacillus* spp. following raffinose fermentation is consistent with previous reports on the prebiotic effects of raffinose and other non-digestible oligosaccharides [2,3,4]. However, our culturomics analysis revealed an intriguing functional discrepancy. Despite their high abundance in fecal samples, individual *Bifidobacterium* strains exhibited only limited raffinose-degrading capacity, whereas *L. reuteri* subsp. *reuteri* R14-1 and *L. mucosae* R14-4 demonstrated the highest degradation efficiency. This observation highlights the importance of moving beyond compositional analyses to direct functional characterization at the strain level. The reclassification of *Lactobacillus reuteri* to *Limosilactobacillus reuteri* in 2020 reflects its phylogenetic and functional distinctiveness [17], and our findings further support its role as a key player in raffinose metabolism. Notably, the ability of *L. reuteri* to utilize raffinose has been linked to the presence of specific glycosyl hydrolases and transport systems [29,30], which may explain its superior performance compared to *Bifidobacterium* species in our study.

Mechanistically, previous studies have demonstrated that *L. reuteri* preferentially utilizes raffinose via secondary transporters, and that this carbohydrate utilization profile is conserved across strains of different origins and lineages rather than being restricted to a particular subspecies [29,30]. Nevertheless, the substantially higher degradation efficiency observed for subsp. *reuteri* R14-1 compared to other isolates in our study suggests that strain-level differences in the expression or regulation of these conserved transport systems may determine the functional potency of raffinose utilization, even when the genetic capacity is broadly distributed across the species. While the genetic capacity for raffinose utilization appears to be conserved across *L. reuteri* strains [29,30], the superior degradation efficiency of subsp. *reuteri* R14-1 points to strain-specific functional differences that warrant further mechanistic exploration.

The metabolic reprogramming of *L. reuteri* subsp. *reuteri* R14-1 in response to raffinose represents a central finding of this study. Untargeted metabolomics revealed that raffinose induced a distinct metabolic landscape compared to glucose, with 290 metabolites significantly upregulated. Among these, oxyphencyclimine and 1-kestose—both recognized for their anti-inflammatory and antioxidant properties—were markedly elevated [20,21,22]. KEGG enrichment analysis further revealed coordinated remodeling of pathways involved in nucleotide metabolism, alanine/aspartate/glutamate metabolism, and glutathione metabolism. Glutathione, a key antioxidant in bacterial and host cells, plays a critical role in mitigating oxidative stress and maintaining cellular homeostasis [31,32]. The enrichment of glutathione metabolism in the raffinose group suggests that this carbon source may promote a metabolic state that enhances stress resilience and bioactive compound production. These findings align with the well-documented immunomodulatory properties of *L. reuteri* and provide a mechanistic basis for its probiotic potential in the context of prebiotic supplementation [33,34,35].

The present study has several implications for the development of personalized nutrition and synbiotic interventions. The observed interindividual variation in raffinose fermentation underscores the need for microbiome-based stratification in prebiotic efficacy studies. Furthermore, the identification of *L. reuteri* subsp. *reuteri* R14-1 as an efficient raffinose degrader capable of producing anti-inflammatory metabolites positions this strain as a promising candidate for synbiotic formulations. Synbiotics, defined as combinations of prebiotics and live microorganisms that confer health benefits [36,37,38,39], represent an emerging strategy for targeted modulation of the gut microbiota [40,41,42]. Given that raffinose selectively promotes the growth of *L. reuteri* while also serving as a substrate for its production of bioactive metabolites, the pairing of raffinose with *L. reuteri* R14-1 may offer synergistic effects beyond those of either component alone. However, it is important to emphasize that our in vitro findings provide a mechanistic rationale rather than a guarantee of in vivo efficacy. Successful colonization, persistence, and competitive fitness of *L. reuteri* subsp. *reuteri* R14-1 within the complex gut ecosystem are not assured and depend on multiple factors including strain-specific traits, host immune status, and baseline microbiota composition. This is a standard consideration in the rational design of synbiotic interventions. Future studies should evaluate this synbiotic combination in vivo, assess its impact on gut barrier function and inflammation, and explore its potential applications in metabolic and inflammatory diseases. Additionally, while our in vitro approach enabled high-resolution functional characterization, further investigations using humanized gnotobiotic models or clinical trials will be necessary to validate the translational relevance of our findings.

Several limitations of this study should be acknowledged. First, the sample size of 16 healthy donors, while sufficient to detect pronounced interindividual differences in raffinose degradation and to support subsequent strain-level functional analyses, is modest for a study of interindividual variability. Many similar investigations in the literature include 20–40 or more donors. Our findings should therefore be considered exploratory, and the generalizability of the observed variation patterns to broader populations requires validation in larger cohorts.

Second, our in vitro batch fermentation system, while enabling controlled functional screening and metabolomic profiling, does not fully recapitulate the complex ecological interactions present in the native gut environment. Third, although we identified *L. reuteri* subsp. *reuteri* R14-1 as a highly efficient in vitro degrader, its ecological role and functional impact within the intact human gut microbiome remain to be established through in vivo studies using gnotobiotic models or human intervention trials.

Fourth, detailed host covariate data, including BMI, habitual dietary fiber intake, and medical history, were not collected for all participants. While all donors were recruited from the same geographic region (Qingdao, China), which reduces environmental heterogeneity, the absence of these covariates limits our ability to identify the specific host factors that drive the observed interindividual variation. Future studies should incorporate comprehensive donor phenotyping to disentangle the relative contributions of host genetics, diet, and lifestyle to prebiotic fermentation outcomes.

Fifth, our experimental design employed only one fermentation run per donor sample, without technical replicates. This design does not allow for formal separation of biological variability from technical variability. However, several precautions were taken to minimize technical noise: all fermentations were conducted simultaneously in the same anaerobic chamber using media from the same batch and a standardized inoculum preparation protocol. More importantly, the core conclusions of this study are robust to the absence of technical replicates, as they are based on within-donor comparisons (pre- vs post-fermentation), functional screening of isolated strains under controlled conditions, and metabolomic profiling with six independent biological replicates. Nonetheless, future investigations should incorporate technical replicates to more rigorously separate biological and technical sources of variation and to confirm the reproducibility of the observed individual differences in raffinose degradation.

Sixth, our in vitro batch fermentation system, while enabling controlled functional screening and metabolomic profiling, does not fully recapitulate the complex ecological interactions present in the native gut environment. In addition, the VI medium used in this study is rich in peptides and contains Tween 80, which may inherently bias community composition toward *Bifidobacterium* spp. and *Lactobacillus* spp. Consequently, the raffinose-induced enrichment of these taxa observed in our system may overestimate their in vivo competitive advantage. However, this medium-driven bias does not affect our subsequent strain-level functional assessments, as all isolated strains were screened under identical culture conditions, enabling direct comparison of their relative degradation capacities. Future studies using chemically defined media or more complex gut-simulating models could help clarify the extent to which these biases influence community-level fermentation outcomes.

Seventh, the metabolomic findings presented in this study, while revealing distinct metabolic reprogramming of *L. reuteri* subsp. *reuteri* on raffinose, should be interpreted with caution. All metabolite identifications were based on untargeted MS/MS spectral matching without further validation using authentic standards or enzymatic assays. For example, we reported oxyphencyclimine as an upregulated metabolite, but upon critical re-evaluation, we acknowledge that this synthetic pharmaceutical compound might be a false positive arising from database misannotation or the presence of isomeric compounds with similar mass spectral features. Similarly, while 1-kestose is a biologically plausible metabolite given the known fructosyltransferase machinery of *L. reuteri*, our identification of this compound remains putative without genomic or enzymatic confirmation. These examples underscore the inherent limitations of untargeted metabolomics, where database-dependent identifications should be considered hypothesis-generating rather than definitive. Future studies should employ targeted metabolomics with reference standards, enzymatic assays, and genomic screening to validate the production of specific bioactive metabolites by *L. reuteri* subsp. *reuteri* R14-1. Furthermore, while we identified 1-kestose as a putatively upregulated metabolite with documented bioactivities in the literature, our study does not provide any functional evidence of host interaction, bioavailability, or concentration-dependent effects. Metabolomic detection alone, particularly in an in vitro system devoid of host cells, immune components, and mucosal barriers, cannot be extrapolated to physiological relevance or clinical benefit. We caution readers against interpreting our metabolomic findings as proof of health-promoting properties. Rather, these observations provide a rational basis for future hypothesis-driven investigations that combine targeted metabolomics with functional bioassays and in vivo validation. Despite these limitations, the integrative culturomics–metabolomics pipeline established here provides a robust framework for strain-level functional dissection of prebiotic–microbe interactions and offers a rational basis for future targeted synbiotic development.

## 5. Conclusions

In conclusion, this study provides a comprehensive, strain-level resolution of raffinose fermentation by the human gut microbiota. Using an integrative approach combining in vitro anaerobic culture, 16S rRNA gene sequencing, culturomics, and untargeted metabolomics, we demonstrated that raffinose degradation exhibits substantial interindividual variation, underscoring the personalized nature of prebiotic utilization. Although raffinose selectively enriches *Bifidobacterium* spp. and *Lactobacillus* spp. at the community level, functional characterization at the strain level revealed that *L. reuteri* subsp. *reuteri* R14-1, rather than *Bifidobacterium* species, is the most efficient degrader. Notably, raffinose induced a distinct metabolic reprogramming in this strain, leading to the production of bioactive metabolites with anti-inflammatory properties, including oxyphencyclimine and 1-kestose, alongside coordinated remodeling of nucleotide and amino acid metabolism. These findings establish a mechanistic framework for understanding how raffinose functions as a prebiotic and highlight the importance of moving beyond compositional analyses toward strain-level functional characterization. The identification of *L. reuteri* subsp. *reuteri* R14-1 as a key metabolic hub linking prebiotic consumption to bioactive metabolite production positions this strain as a promising candidate for synbiotic development. Future studies should evaluate the in vivo efficacy of the raffinose–*L. reuteri* synbiotic combination in modulating gut health, inflammation, and metabolic homeostasis, thereby translating these mechanistic insights into actionable strategies for personalized nutrition and therapeutic intervention.

## Data Availability

The original contributions presented in this study are included in the article/Supplementary Materials. Further inquiries can be directed to the corresponding author.

## Supplementary Materials

The following supporting information can be downloaded at: https://www.mdpi.com/article/10.3390/foods15152649/s1, Figure S1. Alpha diversity of gut microbiota before and after in vitro raffinose fermentation. Comparison of Shannon index (A), Simpson index (B), observed species (C), Chao1 index (D), and Ace index (E) between baseline (before fermentation) and post-fermentation samples. *** *p* < 0.001. Figure S2. Summary of raffinose-degrading bacterial strains isolated from human fecal samples. (A) Pie chart showing the species-level distribution and relative proportions of 204 isolated bacterial strains. (B) Heatmap illustrating the abundance and distribution of isolated bacterial species across 16 individual fecal samples. Each column represents one donor, and the color intensity indicates the number of colonies isolated for each species. Table S1. Basic information of the 16 healthy volunteers.

## Abbreviations

The following abbreviations are used in this manuscript:

OD	Optical density
TLC	Thin layer chromatography
SCFAs	Short-chain fatty acids
KEGG	Kyoto Encyclopedia of Genes and Genomes
CFUs	Colony forming units
HPLC	High-performance liquid chromatography
MEGA	Molecular Evolutionary Genetics Analysis
PCA	Principal component analysis
PLS-DA	Partial least squares-discriminant analysis
