# Supplementary material for: Interindividual Variability in Raffinose Fermentation by Human Gut Microbiota and Metabolomic Signature of a High-Efficiency In Vitro Degrader, Limosilactobacillus reuteri subsp. reuteri
Source: Foods. 2026 Jul 28;15(15):2649. doi: 10.3390/foods15152649 (PMC13464821; doi:10.3390/foods15152649)
Supplement: Supplementary file 1 [file foods-15-02649-s001.zip › foods-4449462-supplementary.pdf]

*Article*

# Interindividual Variability in Raffinose Fermentation by Human Gut Microbiota and Metabolomic Signature of a High-Efficiency In Vitro Degrader, *Limosilactobacillus reuteri* subsp. *reuteri*

Wei Dai [1,†], Min Quan [1,†], Guangli Yu [1,2,3] and Qingsen Shang [1,2,3,*]

[1] *Key Laboratory of Marine Drugs of Ministry of Education, Shandong Key Laboratory of Glycoscience and Glycotherapeutics, School of Medicine and Pharmacy, Ocean University of China, Qingdao 266003, China*

[2] *Laboratory for Marine Drugs and Bioproducts, Qingdao Marine Science and Technology Center, Qingdao 266237, China*

[3] *Marine Biomedical Research Institute of Qingdao, Qingdao 266071, China*

* Correspondence: shangqingsen@ouc.edu.cn

† These authors contributed equally to this work

## Supplementary Figures and Tables

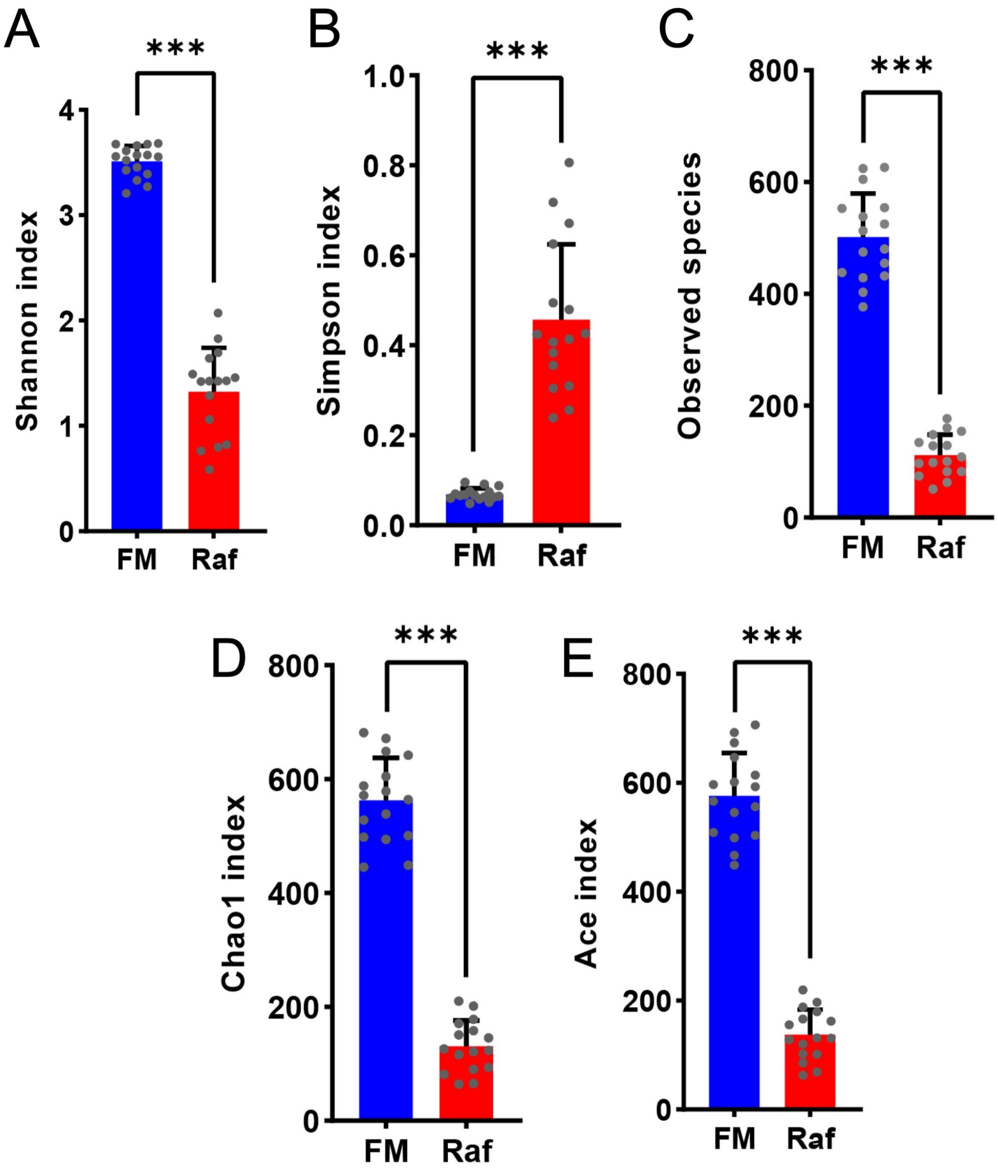


**Figure S1.** Alpha diversity of gut microbiota before and after *in vitro* raffinose fermentation. Comparison of Shannon index (A), Simpson index (B), observed species (C), Chao1 index (D), and Ace index (E) between baseline (before fermentation) and post-fermentation samples. ***$P < 0.001$.

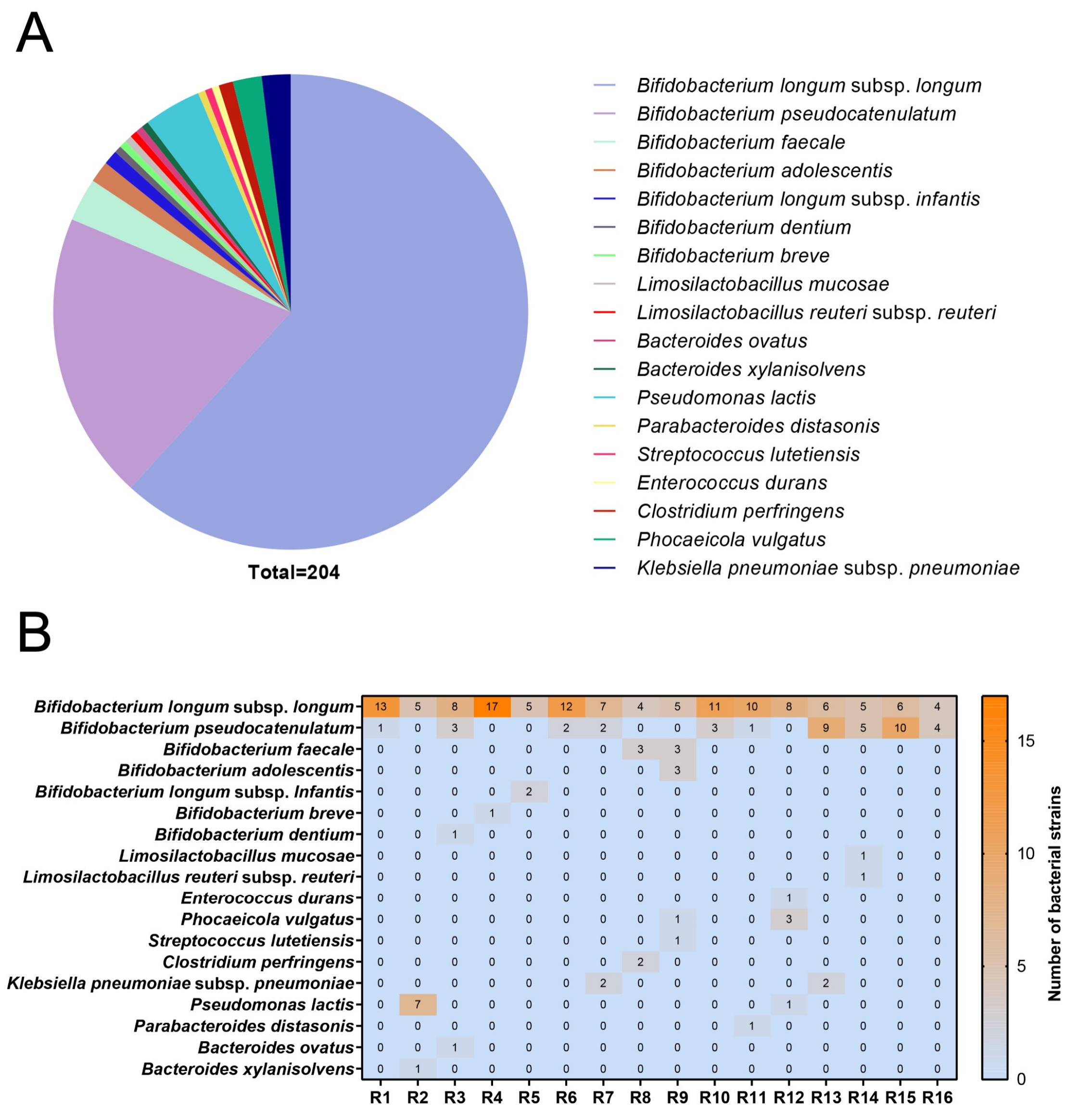


**Figure S2.** Summary of raffinose-degrading bacterial strains isolated from human fecal samples. (A) Pie chart showing the species-level distribution and relative proportions of 204 isolated bacterial strains. (B) Heatmap illustrating the abundance and distribution of isolated bacterial species across 16 individual fecal samples. Each column represents one donor, and the color intensity indicates the number of colonies isolated for each species.

**Table S1.** Basic information of the 16 healthy volunteers.

| Volunteer ID | Gender | Age |
|---|---|---|
| R1 | F | 24 |
| R2 | M | 24 |
| R3 | F | 25 |
| R4 | F | 26 |
| R5 | F | 24 |
| R6 | M | 26 |
| R7 | F | 24 |
| R8 | M | 24 |
| R9 | M | 25 |
| R10 | F | 23 |
| R11 | M | 23 |
| R12 | F | 23 |
| R13 | M | 23 |
| R14 | F | 24 |
| R15 | M | 26 |
| R16 | F | 4 |
